# Wearable rehabilitation wristband for distal radius fractures

**DOI:** 10.3389/fnins.2023.1238176

**Published:** 2023-09-14

**Authors:** Qing Zha, Zeou Xu, Xuefeng Cai, Guodong Zhang, Xiaofeng Shen

**Affiliations:** ^1^School of Biomedical Engineering (Suzhou), Division of Life Sciences and Medicine, University of Science and Technology of China, Hefei, China; ^2^Suzhou Institute of Biomedical Engineering and Technology, Chinese Academy of Science, Suzhou, China; ^3^Suzhou TCM Hospital Affiliated to Nanjing University of Chinese Medicine, Suzhou, China

**Keywords:** distal radius fracture, thin film pressure sensor, rehabilitation training action recognition, autoencoder, SVM

## Abstract

**Background:**

Distal radius fractures are a common type of fracture. For patients treated with closed reduction with splinting, a period of rehabilitation is still required after the removal of the splint. However, there is a general lack of attention and low compliance to rehabilitation training during this period, so it is necessary to build a rehabilitation training monitoring system to improve the efficiency of patients’ rehabilitation.

**Methods:**

A wearable rehabilitation training wristband was proposed, which could be used in the patient’s daily rehabilitation training scenario and could recognize four common wrist rehabilitation actions in real-time by using three thin film pressure sensors to detect the pressure change curve at three points on the wrist. An algorithmic framework for classifying rehabilitation training actions was proposed. In our framework, an action pre-detection strategy was designed to exclude false detections caused by switching initial gestures during rehabilitation training and wait for the arrival of the complete signal. To classify the action signals into four categories, firstly an autoencoder was used to downscale the original signal. Six SVMs were then used for evaluation and voting, and the final action with the highest number of votes would be used as the prediction result.

**Results:**

Experimental results showed that the proposed algorithmic framework achieved an average recognition accuracy of 89.62%, an average recognition recall of 88.93%, and an f1 score of 89.27% on the four rehabilitation training actions.

**Conclusion:**

The developed device has the advantages of being small size and easy to wear, which can quickly and accurately identify and classify four common rehabilitation training actions. It can easily be combined with peripheral devices and technologies (e.g., cell phones, computers, Internet) to build different rehabilitation training scenarios, making it worthwhile to use and promote in clinical settings.

## 1. Introduction

Distal radius fractures are a common type of fracture in adults, accounting for approximately 17.5% of all fracture types (Ochen et al., 2020). Fracture treatment is divided into surgical and non-surgical treatment (He et al., 2020). There is a significant difference in the recovery of physical skeletal indicators between the two different treatments, but there is no significant difference in the functional recovery of the wrist joint, so patients mostly consider non-surgical treatment modalities first (He et al., 2020). A period of rehabilitation after the removal of the cast or splint plays a major role in the recovery of joint function (Badwaik et al., 2021). However, there is a common phenomenon that patients do not pay attention to rehabilitation training, have poor compliance, and have irregular training movements (Bhan et al., 2021). Therefore, it is important to build a rehabilitation training assistance monitoring system so that the physician can supervise the patient promptly and increase the patient’s self-motivation for rehabilitation training.

Rehabilitation training assistance monitoring system for the patient’s terminal equipment puts forward the requirements of physiological signal acquisition, rehabilitation training action recognition and evaluation, of which rehabilitation training action recognition is the main research direction, because this technology is the key to build a bridge of joint supervision between doctor and patient for distal radius fracture, and a large number of researches have existed for this task. The means of rehabilitation training action recognition fall into two broad categories, namely computer vision (CV) based and sensor based approaches (Zhu et al., 2019). The computer vision-based approach acquires raw image information through a vision-based sensor, then performs extraction of low-level features such as human joint positions, and after encoding and representing the feature data, it performs a number of tasks such as kinematic parameter comparisons, postural recognition, and clinical scoring (Debnath et al., 2022). Many image processing and machine learning techniques have been applied to these studies (Keskin et al., 2012; Tang et al., 2014; Sinha et al., 2016; Wan et al., 2019; Francisco and Rodrigues, 2022; Shen and Lu, 2022; Sun et al., 2022), which typically use single or multiple RGB or depth cameras as image acquisition units and analyze the images or videos to identify static gestures or motion flows within them (Hellsten et al., 2021). Although CV-based systems have the advantages of simple equipment and low cost, vision algorithms are inevitably accompanied by the shortcomings of being highly influenced by occlusions and light. More critically, CV-based systems have a single source of data (only image pixel information) and therefore lack the means to robustly monitor the patient’s physiological parameters (e.g., pressure on the affected area), yet the tightness of the splints used for immobilization is an important influence on the outcome of fracture rehabilitation (Li et al., 2021). Therefore, the lack of capability of CV-based systems in this area is the greatest drawback compared to sensor-based rehabilitation training systems.

Sensor-based rehabilitation training devices have unique advantages due to their ability to detect multimodal physiological parameters directly or indirectly. These systems place multiple types of sensors on different carriers, and the data is collected and analyzed by a central processor (Nascimento et al., 2020; Yadav et al., 2021). Common sensors used in wrist rehabilitation systems include pressure sensors (Zhang et al., 2019; Atitallah et al., 2020; Guo et al., 2021; Pierre Claver and Zhao, 2021; Xu et al., 2021), surface electromyographic(sEMG) sensors (Prakash et al., 2019; Cheng et al., 2021; Dong et al., 2021; Moin et al., 2021; Copaci et al., 2022; Jeong et al., 2022), inertial sensors (Kim et al., 2019; Weygers et al., 2020; Bilius et al., 2023), and specialized sensors (e.g., acoustic sensors (Xiao et al., 2022), strain sensors (Gao et al., 2023). Currently, the main researched rehabilitation training devices usually include multiple sensors to achieve multimodal and more accurate training movement analysis, and the main presentation of wrist movement recognition devices is the rehabilitation glove. For example, Copaci et al. proposed a gesture classification algorithm for rehabilitation training gloves based on surface EMG signals, which is based on Bayesian neural networks, pattern recognition networks, and hierarchical recurrent networks, and allows users to retrain the algorithm at any time with their own surface EMG gesture data, with a recognition accuracy of up to 98.7% for six types of gestures (Copaci et al., 2022). Li et al. (2023) developed a set of multimodal sensor gloves for hand kinematics learning in Parkinson’s patients, which used flexible bending sensors to detect finger curvature information, thin-film pressure sensors to measure changes in hand muscle strength, and an inertial navigation system to detect acceleration signals, and carried out a number of evaluations of finger dexterity, muscle strength, and other assessments based on multiple signal processing algorithm. Meng et al. developed a personalized and safe soft glove for rehabilitation training, which uses a pneumatic actuator module to provide active rehabilitation training for patients and acquires finger bending information based on bending sensor and air-pressure sensor. The system they developed included three modes of rehabilitation training to meet the rehabilitation requirements of patients with multiple hand dysfunctions (Meng et al., 2023). Since the rehabilitation glove provides a stable platform for sensor placement, it is particularly suitable for multimodal hand movement analysis (and, of course, wrist rehabilitation). Moreover, due to the large number of sensors it can deploy, accurate acquisition of finger bending information and hand posture information can be easily realized, and thus hand movement recognition based on such information can be easily achieved with good results. Compared to vision-based approaches, sensor-based rehabilitation assistance devices have some significant advantages, such as the accuracy of physiological information acquisition and the minuteness to environmental interference. In addition to rehabilitation training gloves, some special and novel rehabilitation assistance devices were also presented. For example, Han et al. (2022) proposed a cylindrical device based on a passive sensing layer called smart skin, which estimates the grip force by the change in the shape of the colored liquid in the subtle channels during gripping. Wong et al. (2021) developed a finger-worn capacitive sensor system that utilizes capacitance changes due to different hand movements for gesture classification tasks.

However, the main applications of these studies on hand rehabilitation assistance systems are for stroke and Parkinson’s patients, and these application scenarios do not limit the pressure at the radius. In addition to proper rehabilitation, patients with distal radius fractures should also ensure that the pressure on the affected area of the radius is within the appropriate range, otherwise excessive splint pressure will likely bring about various syndromes as a result of vascular compression of the affected area, while too little pressure on the splint may result in secondary dislocation of the fracture. Therefore, rehabilitation equipment for patients with distal radius fractures should have the ability to monitor skin pressure on the affected area in addition to supervised rehabilitation. At the same time, the variety and number of sensors deployed in rehabilitation training gloves [e.g., the data glove developed by Bin Fang et al. carries 36 inertial measurement units (Fang et al., 2019)] inevitably brings about an increase in cost, which no doubt increases the burden of treatment for patients. To address these issues, we designed a wearable rehabilitation wristband that used only three thin-film pressure sensors as the components for skin pressure signal acquisition. The thin-film pressure sensors are inexpensive to produce and can be mounted non-invasively between the patient’s skin and the splint. The conversion of pressure data and the recognition of rehabilitation training actions are realized through the topmost control box, and the relevant information will be sent to the matching cell phone APP for display and storage. Compared to rehabilitation gloves, our devices are extremely low-cost and allow effective monitoring of splint tightness. The main contributions of this paper are as follows:

1. We proposed a wearable rehabilitation training wristband for distal radius fractures, which provided rehabilitation training actions recognition and detection functions, and opened up a Bluetooth interface that allowed simple connection to computers, cell phones, and other upper computers and the development of a variety of rehabilitation training software.
2. A rehabilitation training action classification algorithm based on an autoencoder and SVM classifier was designed, which could run on a microcontroller and classified action signals quickly and accurately.
3. An action signal pre-detection strategy was proposed to determine whether the window signal was a complete action signal, which reduced the false detection rate of the algorithm.

## 2. Materials and methods

### 2.1. Materials

#### 2.1.1. Thin film pressure sensor

**Figure 1:**
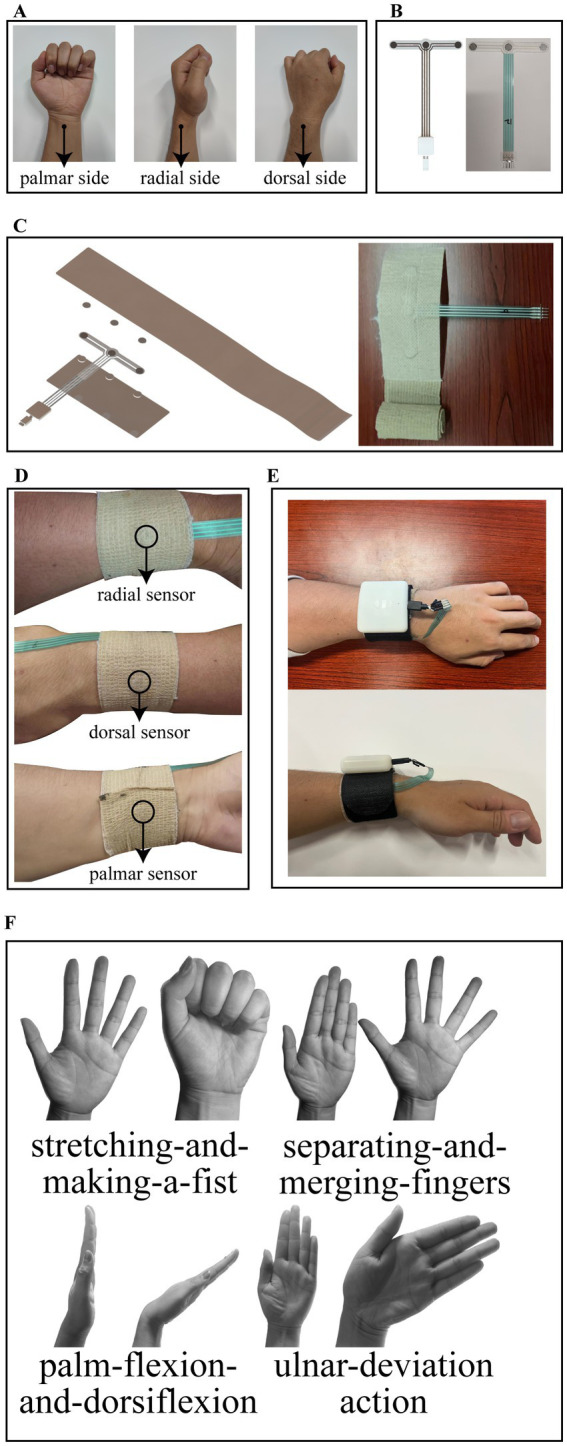
**(A)** Location of the collection points. **(B)** Design structure and object diagram of the thin film pressure sensor. **(C)** Structural schematic and object diagram of the inner bandage core. **(D)** Schematic of the bandage core after completion of wear. **(E)** Schematic of the entire system after wearing. **(F)** Rehabilitation training standardized actions including stretching-and-making-a-fist, separating-and-merging-fingers, palm-flexion-and-dorsiflexion, and ulnar-deviation action.

Thin-film pressure sensors are used to detect three channels of pressure on the palmar, radial and dorsal sides of the wrist. Hua et al. (2018) performed a biomechanical finite element analysis of the stress distribution on the arm for three common types of splints, and their stress analysis results showed that all three types of splints produced the greatest stresses in the vicinity of the radial stem eminence at a one-week location, but the absolute values of the stresses were different. Therefore, we followed the wrist force characteristics of splinting and chose the location of the radial collection point to be 1–2 cm from the malleolus on the lateral side of the radius, which is located near the distal radius fracture point and the maximal stress of the splint, so as to monitor the lateral force on the fracture point in an effective and obvious way. The dorsal and palmar collection points are centrally located on the dorsum and palm of the hand, respectively, and are on the same circumference as the radial collection point. The location of the collection points is shown schematically in Figure 1A.

A three-channel thin-film pressure sensor designed by our own structure is used as the detection element of the wristband, and presents a T-shaped structure. On the crossbeam of the T, three pressure-sensitive zones are distributed from left to right for the above-mentioned palmar, radial, and dorsal pressure detection. On the arm of the T, there are four copper wires leading out and connected to the control box through the MicroUSB interface, one of which is connected to one of the pins of the three pressure-sensitive zones to form a common terminal. The T-shaped structure design avoids the problem of cable stacking when wearing the wristband. The design structure and object diagram of the thin film pressure sensor are shown in Figure 1B.

#### 2.1.2. Rehabilitation wristband

The rehabilitation wristband is composed of an inner bandage core and an outer magic stick layer. The inner bandage core consists of two layers of medical bandages and film sensors pressed together, with the upper layer of bandages for the elastic self-adhesive bandages, longer length, for wearing around the fixed, and the lower layer of bandages for inelastic bandages. Both sides of the thin-film sensor are in contact with the upper and lower bandages by circular silicone pads with a thickness of 1 mm. The structural schematic of the inner bandage core is shown in Figure 1C along with its object diagram. The outer layer of the magic stick has rigid fibers on one side to provide a stable force platform for the pressure sensor and a magic stick on the other side to hold the entire structure in place. Our circuit board is enclosed in a control box, which is fixed above the outer magic stick and connected to the thin film sensors through an electrical interface. When worn, the radial sensor in the inner bandage core is first positioned at the location of the radial collection point described above, followed by stroking the wristband both palmarly and dorsally so that the wristband is wrinkle-free and fits completely around this part of the wrist, and finally the self-adhesive bandage of the wristband is wrapped around the wrist for 1 week and compacted to hold it in place. The schematic of the bandage core after completion of wear is shown in Figure 1D. Subsequently, the rigid side of the Velcro was attached to the inner bandage core, wrapped around for a week and then secured by a carabiner, and finally, the sensor interface was plugged into the control box. The schematic of the entire system after wearing is shown in Figure 1E. As you can see, the system we designed is simple in structure and compact in size. It should be noted that the remaining portion of the outer magic stick layer can be easily embedded in various splint systems (e.g., plaster splints, small splints, thermoplastic splints, etc.) as a splint pressure status monitoring terminal during the splint fixation period, which is an important use of this system beyond the description herein.

#### 2.1.3. Control box circuit

The control box circuit is composed of four parts: a power supply circuit, STM32 microcontroller minimum system, pressure acquisition system, and BLE Bluetooth transmission circuit. The power supply circuit is used to charge the lithium battery and to regulate the input voltage of the lithium battery to the voltage required by other chips. It uses the PW5410A charge pump chip to regulate the input voltage of the lithium battery to 5 V, which is then stepped down to 3.3 V by the PW6566 LDO chip. 5 V is supplied to the operational amplifier in the system, and 3.3 V is supplied to the STM32 microcontroller, BLE Bluetooth transceiver module, and other chips. The STM32 microcontroller minimal system is used to detect the analog signal output from the pressure acquisition system, detect the transceiver signal of the Bluetooth module and run the classification algorithm of the rehabilitation action signal, which uses its own ADC peripheral to convert the analog voltage to digital, and uses the BLE Bluetooth module to send the detected action signal or receive the control signal from the upper computer. The pressure acquisition system consists of three Wheatstone bridges cascaded with differential amplification circuits, each of which is used to detect the pressure sensor information of one channel, and the output voltage of the pressure acquisition system is 0–3.3 V, which is received by the STM32 microcontroller. The BLE Bluetooth transmission module serves as a wireless communication medium between the control box and the upper computer, automatically converting serial signals and Bluetooth RF signals to each other, which simplifies the system development difficulty. The circuit system structure and module circuits are shown in Supplementary Figure S1.

#### 2.1.4. Rehabilitation training action specification

Although it is now clinically recognized that appropriate functional exercises have a positive effect on the rehabilitation of distal radius fractures, there is no uniform standard for the details of the actions of rehabilitation training (Østergaard et al., 2021). Often hospitals in different areas will prescribe different rehabilitation exercises to patients. For example, in a clinical study of functional exercises based on cast immobilized patients conducted by Reid et al. (2020), patients were asked to perform palmar flexion and dorsiflexion and fist clenching for the wrist joint. Arora and Naqvi (2022) in a clinical validation trial of a leap motion tracking device had patients perform five movements: fingers flexion and extension, flexion and extension of the thumb, wrist radial and ulnar deviation, forearm pronation and supination and wrist flexion and extension. The results of the experiment verified the effectiveness of these rehabilitation exercises. Some other forms of rehabilitation training methods such as wrist rotation (Huang et al., 2019) and grip training (Quadlbauer et al., 2020) were also used by some organizations. We considered the available literature and selected the following four typical rehabilitation exercises by the chief physician of the author’s hospital unit (Li et al., 2020), namely, stretching-and-making-a-fist, separating-and-merging-fingers, palm-flexion-and-dorsiflexion, and ulnar-deviation action. For rehabilitation, place the elbow on a flat table and hold the forearm upright in a neutral position. When stretching and making a fist, the palm of the hand is relaxed as the initial state, then the fingers are stretched, followed by a fist clenching as hard as possible, and finally returning to the initial state, which is considered a complete action. When separating and merging fingers, the palm of the hand is initially held with the five fingers together and straightened, then the fingers are separated as far as possible and finally returned to the initial state, which is considered a complete action. When performing palm flexion and dorsiflexion, the palm first maintains the same initial state of separating and merging fingers movement, then the palm tilts forward as far as possible to the side of the palm, followed by the palm tilting backward as far as possible to the dorsal direction, and finally returns to the initial state, which is considered a complete action. When performing the ulnar deviation movement, the palm is first maintained in the same initial state as the separating and merging fingers movement and then tilted towards the ulna as far as possible, and finally returned to the initial state, which is regarded as a complete action. The rehabilitation training standardized action is shown in Figure 1F.

### 2.2. Methods

#### 2.2.1. Thin film pressure sensor calibration

The thin-film pressure sensors we used are resistance-strain sensors, whose resistance decreases gradually as the applied pressure increases. After conversion by the sensor data acquisition circuit, the change in applied pressure will be reflected as a change in the output analog voltage of the circuit, which is converted into a digital quantity by the analog-to-digital conversion module of the microcontroller (hereinafter referred to as the AD value). Since it is not possible to calculate the applied pressure directly from the AD value, we conducted pressure calibration experiments of the sensor with the aim of finding the mapping between the pressure applied to the sensor and the corresponding output AD value.

**Figure 2:**
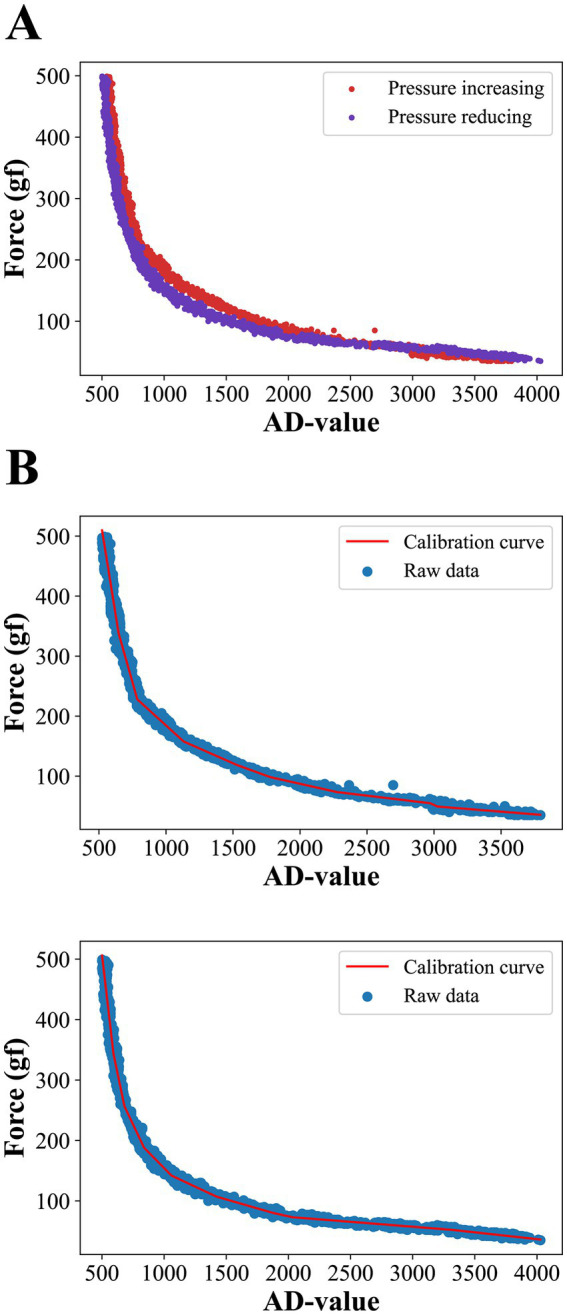
**(A)** Raw data from pressure collection. Due to hysteresis errors, the curves do not coincide exactly during forward and reverse pressure applying. **(B)** Results of fitting forward and backward curves using a 9-segmented linear function.

We first placed the pressure sensor on the pressure calibration platform, which consisted of a push-pull force gauge with a 3 kg range and 1 g resolution and a hand-cranked fixture. The push-pull gauge was fixed on a stationary frame and different pressures were applied to the pressure sensors by turning the rocker, and the AD values of the corresponding channels of the pressure sensor and the pressure measurements of the push-pull gauge were simultaneously collected by our computer. A total of 10 rounds of raw data were collected, with each round collecting 300 sets of data at a frequency of 3 Hz, in which the applied pressure was gradually increased to 500 gf during the first five rounds of collection, and gradually decreased to 30 gf during the last five rounds of collection (not reduced to 0 because the push-pull gauge had a 30 gf pressure dead zone). The acquisition results are shown in Figure 2A, where the red scatter plot represents gradually increasing pressure data and the blue scatter plot represents gradually decreasing pressure data. It can be seen that the thin-film pressure sensor has a significant hysteresis error, which makes the two pressure data not exactly coincide. We used clusters of segmented linear functions to fit the raw data for pressure increase and decrease separately, a process based on the python library pwlf. Both the final curves were divided into 9 folded segments and the fitted curves are shown in Figure 2B. The top and bottom graphs show the fitted curves for the pressure increase and decrease processes, respectively, and the final pressure prediction deployed to the microcontroller was given by the average of the two fitted curves.

#### 2.2.2. Dataset acquisition

**Figure 3:**
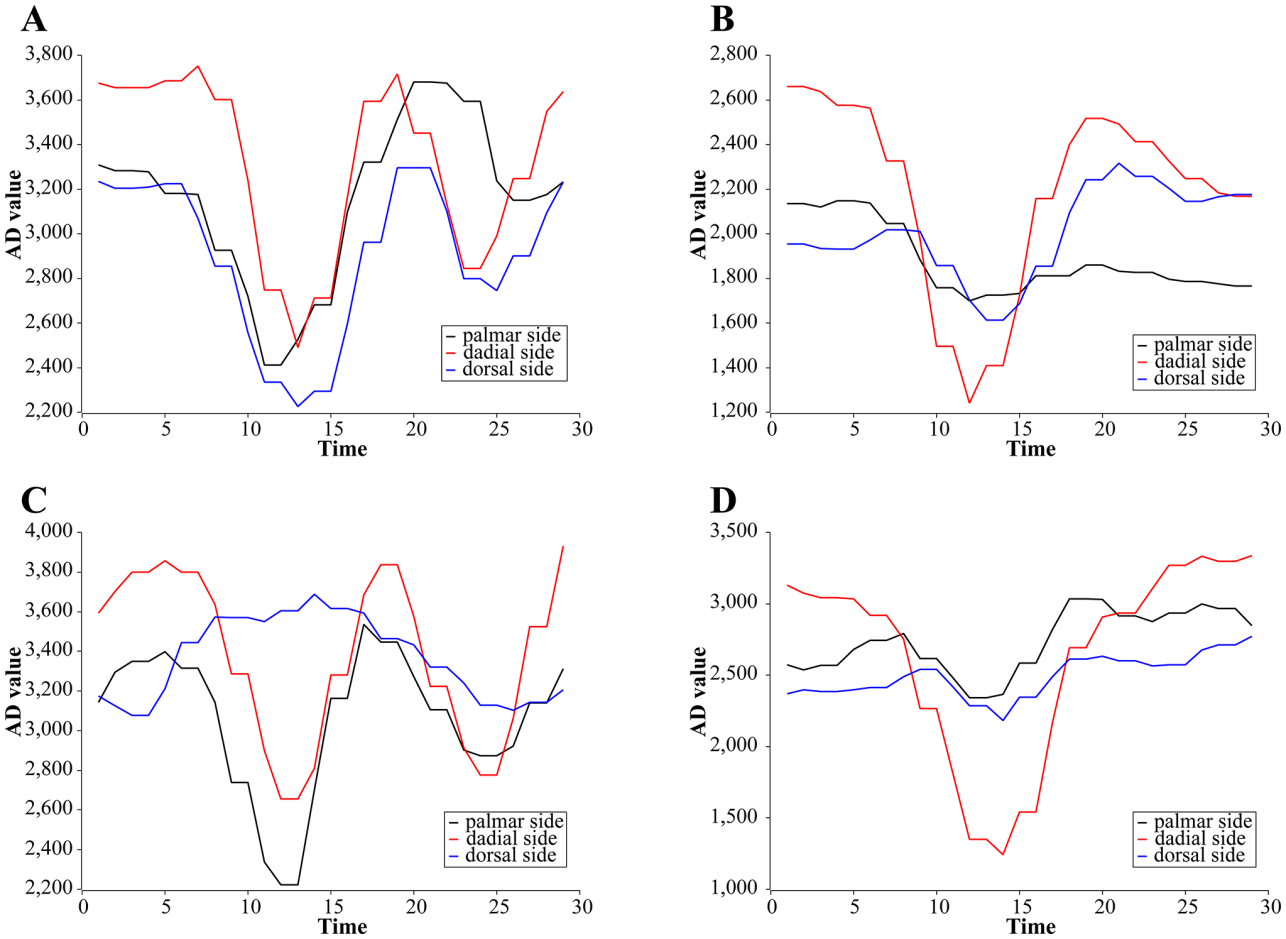
Examples of primary AD change curves for four rehabilitation training actions. **(A)** Stretching-and-making-a-fist. **(B)** Separating-and-merging-fingers. **(C)** Palm-flexion-and-dorsiflexion. **(D)** Ulnar-deviation action.

We use our own QT-based software platform for rehabilitation action acquisition. Five subjects were recruited for training set data collection, with two rounds per subject, and 50 reps of each of the four rehabilitation training actions were collected in turn. The type of raw data collected is the AD value. The sampling frequency of the software platform is 15 Hz, and since the rehabilitation training action is basically completed within 2 s, the number of sample collections is set to 30 times for each group. Thus the data structure of each action sample is $ch_{i}\left(j\right)$, where *i* = 1, 2, 3, *j* = 1, 2, 3,…, 30, and $ch_{i}\left(j\right)$ denotes the j th AD value of the i th channel of the sample. An example of one of the samples we collected is shown in Figure 3.

The initial state pressure varied slightly from subject to subject because the tightness of the rehabilitation wristband was not exactly the same each time the subject was strapped in. We bind an initial reference AD value Refi for the same batch of rehabilitation training for each subject. Firstly, the subject sits at the front of the experimental table and maintains the initial state of stretching and making a fist as required by the rehabilitation training, and then maintains this resting relaxed state and collects a sample, which is called the subject’s resting sample. The resting sample has the same data structure as the action sample, and to distinguish it from the action sample representation, we use $CH_{i}\left(j\right)$ to denote its AD value of it. Refi is precisely calculated by $CH_{i}\left(j\right)$ with the following equation


Refi=∑j=130CHi(j)/30(i=1,2,3)

Subsequently, each action sample data collected by the subject will be transformed into a normalized value $u_{i}\left(j\right)$, where *i* = 1, 2, 3 and *j* = 1, 2, 3,…, 30, which maintains the same data structure as the action sample and is calculated by the following equation


ui(j)=chi(j)Refi

As we can see, $u_{i}\left(j\right)$ is the ratio of the original three-channel AD value of this action sample to its bound initial reference AD value for each channel, which can reduce the impact of data variability caused by wearing rehabilitation wristbands with different tightness to a certain extent and improve the robustness and generalization ability of the system. These data are subsequently arranged in one dimension to form the final input data x(k) of our designed classification algorithm, which is represented by the following equation


$$x\left(k\right)=u_{\alpha }\left(\beta \right)$$

$$\text{Where }\alpha \equiv k\left(\mathrm{mod}\;3,\alpha =1,2,3\right),\beta =\left[\frac{k}{3}\right],k=1,2,3,\ldots ,90.$$

#### 2.2.3. Rehabilitation training movement classification algorithm

Since the input data is a 90-dimensional x(k), this is too many features for an action sample. We first perform dimensionality reduction and feature extraction on it using an autoencoder, which is a mature and effective algorithm for automatically finding features by training an adaptive encoder and decoder to match the original data, where the output of the encoder is the sample feature data we need. Then we classify the encoded data using six SVMs, which, as a binary classification algorithm with linearly divisible samples, improves model generalization by optimizing the hyperplane parameters so that the support vector has the maximum distance from it. Our six SVMs are denoted as $S_{ij}\left(i=1,2,3,4;j=i,\ldots ,4\right)$, which denotes the SVM that classifies action i with action j(the four rehabilitation training action mentioned above are denoted as actions 1–4). The 6 SVMs are given their predicted categories for a sample, and the corresponding category votes are increased by one vote, and finally, the predicted action with the highest number of votes is identified as the final prediction given by the classification algorithm. The structure of the classification algorithm is shown in Supplementary Figure S2.

#### 2.2.4. Action pre-detection strategy

**Figure 4:**
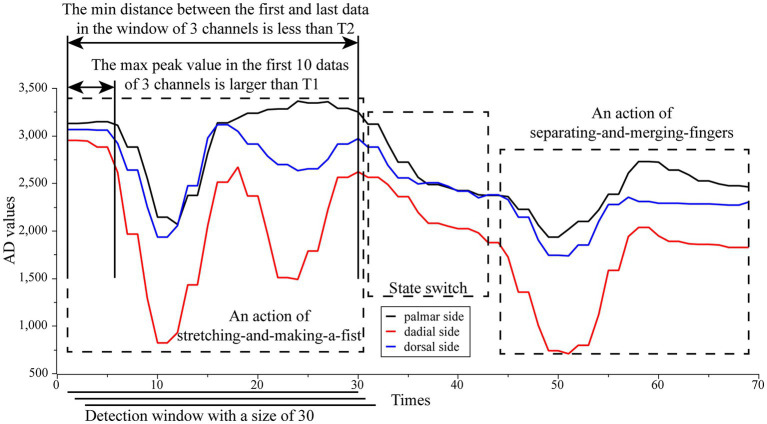
The schematic of the action pre-detection strategy. Only window data that meet the following two conditions will be considered as a rehabilitation training action and input to the classification algorithm for recognition. (1) The maximum peak value of the first 10 data of the three channels of the window is greater than the threshold $T_{1}$, i.e., $\underset{i}{\max}\left\{\underset{j}{\max}\left\{{\text{ch}}_{i}\left(j\right)\right\}-\underset{j}{\min}\left\{{\text{ch}}_{i}\left(j\right)\right\}\right\}>T_{1}$. (2) The minimum head-to-tail AD distance of the three channels of the window is less than the threshold $T_{2}$, i.e., $\underset{i}{\min}\left\{{||\text{ch}}_{i}\left(1\right)-{\text{ch}}_{i}\left(30\right)||\right\}<T_{2}$. Where the threshold values T_1_ and T_2_ are empirical values.

The classification algorithm will be deployed to the STM32 microcontroller to run after the training is completed. According to the input data requirements of the classification algorithm, after the user finishes installing the wristband and prepares the posture for rehabilitation training, the user first keeps the hand relaxed and the upper computer sends initialization instructions to the microcontroller, which continuously acquires 30 sets of three-channel AD values at a frequency of 15 Hz and automatically calculates three average AD values as the initial reference AD value Refi for this round of rehabilitation training. A sliding detection window of size 30 and step 1 is used for real-time detection of rehabilitation training actions. The window divides the current three-channel AD value with Refi at each slide and places it at the end of the window queue, and removes the data at the head of the window queue. If the classification algorithm is propagated forward without discriminating the window data, the action data will be incorrectly classified as another action before it fully enters the window. In addition, the different initial hand postures for different rehabilitation training actions lead to different initial pressures for different actions, which can lead to an upward or downward slope in the data in the window when switching rehabilitation training actions, and if such window data is forward propagated by the classification algorithm, it may also lead to its being judged as one action and thus misclassified. Considering that all rehabilitation movements start and end with similar pressures (this is because all training actions end in the initial posture) and that the three-channel AD values remain essentially constant when the patient is not performing rehabilitation actions, we designed an action data detection strategy that is only window data that meet the following two conditions will be considered as a rehabilitation training action and input to the classification algorithm for recognition. (1) The maximum peak value of the first 10 data of the three channels of the window is greater than the threshold $T_{1}$, i.e., $\underset{i}{\max}\left\{\underset{j}{\max}\left\{{\text{ch}}_{i}\left(j\right)\right\}-\underset{j}{\min}\left\{{\text{ch}}_{i}\left(j\right)\right\}\right\}>T_{1}$. (2) the minimum head-to-tail AD distance of the three channels of the window is less than the threshold $T_{2}$, i.e., $\underset{i}{\min}\left\{{||\text{ch}}_{i}\left(1\right)-{\text{ch}}_{i}\left(30\right)||\right\}<T_{2}$. Where the threshold values $T_{1}$ and $T_{2}$ are empirical values. The first rule ensures that window data is not classified when there is no action, and only window data with sufficiently forward action start points (which ensures that action data is not classified until it enters the window completely) will be classified. The second rule ensures that the window data with a strong change is indeed action data. The schematic of the action pre-detection strategy is shown in Figure 4.

## 3. Results

### 3.1. Pressure calibration results

**Figure 5:**
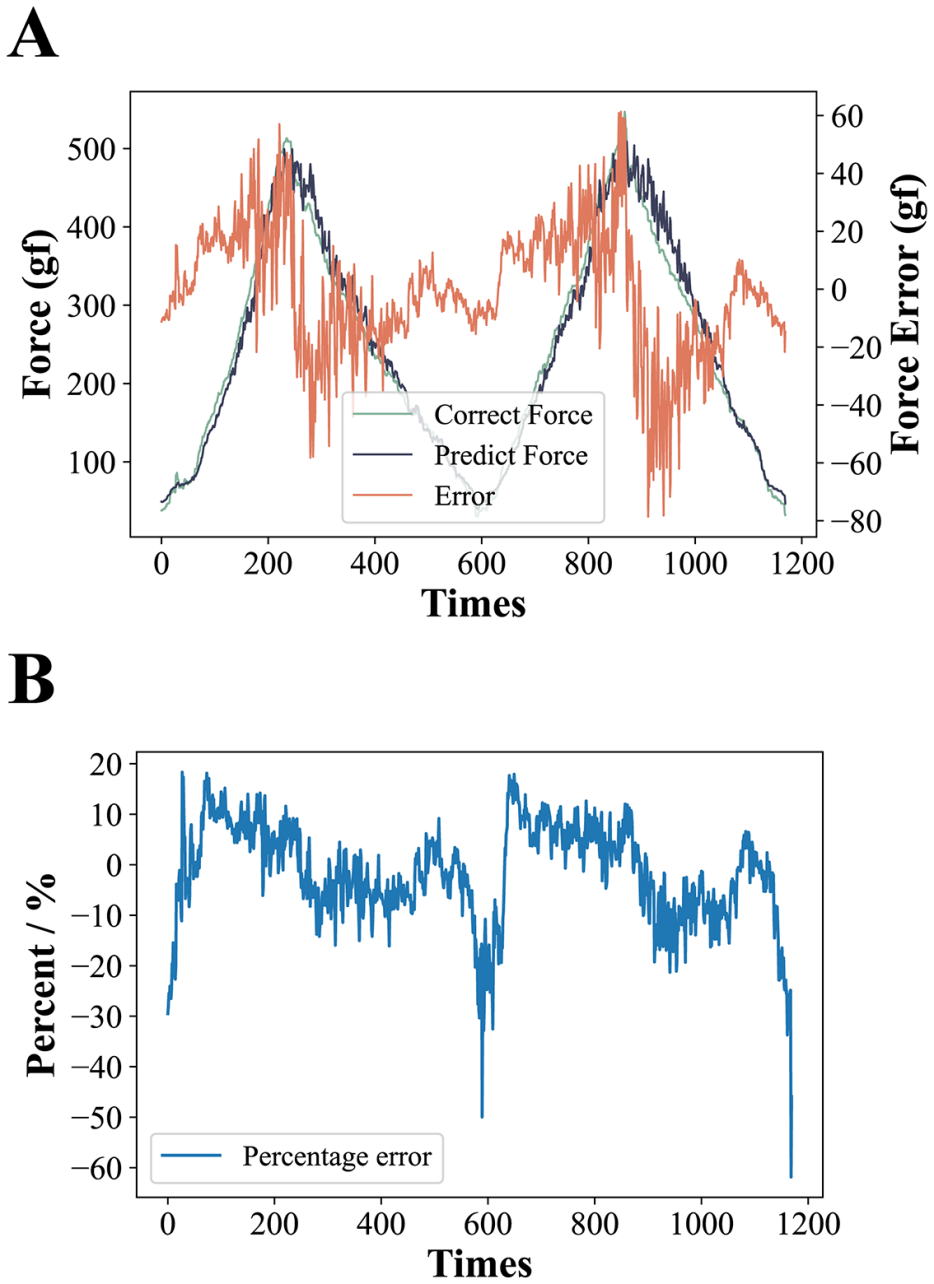
**(A)** Results of pressure calibration tests. The larger error at higher applied pressures is due to the fact that at higher pressures, the larger absolute value of the derivative of the pressure to the AD value results in larger predicted pressure changes from slight AD perturbations. **(B)** The error is expressed as a percentage. Except for individual spikes, the error is basically between ±20%.

We carried out two rounds of pressure calibration test experiments, each round of the first gradually increasing pressure on the sensor to reach the full range and then gradually reduce the pressure, the computer to store the process of pressure prediction of the microcontroller and the real readings of the push-pull gauge. The pressure error is calculated by $f_{\text{error}}=f_{\text{predict}}-f_{\text{true}}$, where $f_{\text{predict}}$ and $f_{\text{true}}$ represent the predicted and true values of the pressure at the same moment in time, respectively, and the results of the two rounds of experiments are spliced together and shown in Figure 5A. The results show that overall the calibration curve seems to work well. The absolute value of $f_{\text{error}}$ becomes progressively larger as the pressure increases. This is due to the fact that the pressure calibration curve has a large absolute value of the derivative of the pressure value to the AD value at higher pressures, resulting in a slight disturbance of the AD at higher pressures causing a large change in the pressure prediction. At one point the absolute value of $f_{\text{error}}$ even exceeded 60gf at pressure values of 400gf or more, but under the range where our equipment is most often used (around 300gf), the absolute value of $f_{\text{error}}$ basically stayed within 20gf. Figure 5B shows the value of $f_{\text{error}}$ divided by $f_{\text{true}}$ at each moment in time. The results show that the pressure calibration error is basically between ±20% throughout the test, except for a very few spikes that reach more than 50%, and these singularities occur at low pressures, which may be due to minor voltage disturbances in the interval between the microcontroller and the push-pull meter data transmission during the data acquisition.

### 3.2. Effect of the number of neurons in the hidden layer and output layer on the verification error of the autoencoder

**Figure 6:**
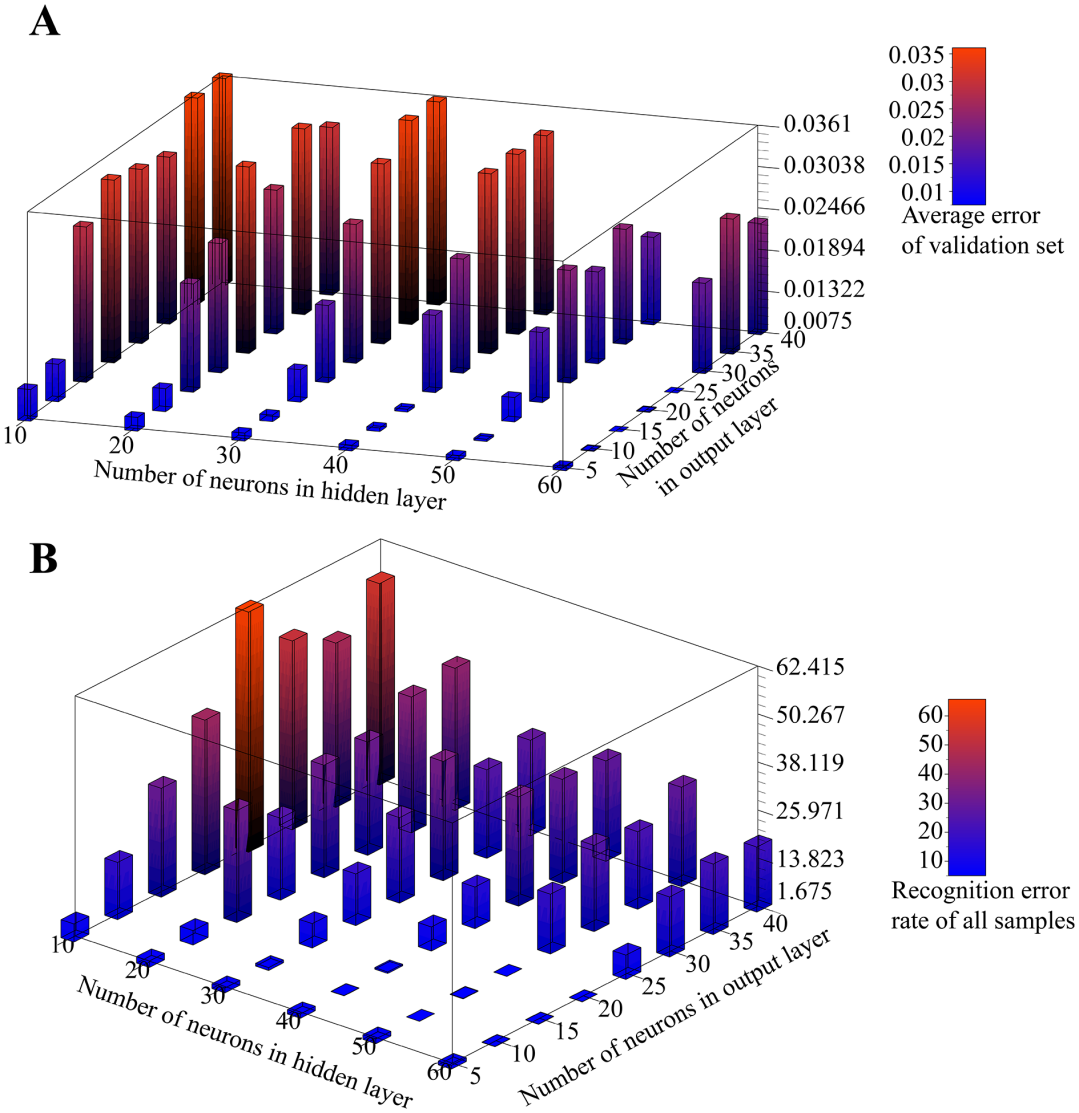
**(A)** Effect of the number of neurons in the hidden layer and output layer on the verification error of the autoencoder. **(B)** Effect of the number of neurons in the hidden layer and output layer on SVM classification.

In order to find the most suitable autoencoder structure for accurate feature extraction of the input data, we tested the effect of different numbers of neurons in hidden layers and output neurons on the validation error of the autoencoder. k-fold cross-validation, as an efficient method to test the performance analysis of networks with different hyperparameters, randomly disturbs the sample set and divides it equally into k subsets. In k times of loops, a different subset is selected as the validation set and the remaining subsets are used as the training set, and the error of the network completed by this loop training on the validation set is evaluated. After k loops, the average of the k test errors is used as a measure of the generalization ability of the network at the current hyperparameter setting. We choose a 10-fold cross-validation approach and let the number of neurons in the hidden layer increase from 10 to 60 in step of 10 and the number of neurons in the output layer increase from 5 to 40 in step of 5. The test results for all combinations are shown in Figure 6A. From the results, it can be seen that with the same number of neurons in the output layer, the average validation error shows a decreasing trend as the number of neurons in the hidden layer decreases, and with the same number of hidden layer neurons, the average validation error shows an increasing trend as the number of output layer neurons increases. Particularly, the network even has difficulty converging when the number of output neurons is larger than the hidden layer. Therefore, with a larger number of hidden layer neurons and a smaller number of output layer neurons, the autoencoder can reduce the dimensionality of the raw data with better accuracy. However, a larger number of hidden layer neurons implies an increase in the number of autoencoder parameters, which increases the execution time for the final deployment of the algorithm to the microcontroller, which is not conducive to real-time processing, while A smaller number of output neurons may lead to a one-sided extraction of features specific to the raw data set by the encoder, which can lead to a reduction in the generalization ability of the algorithm and make it difficult to guarantee its linear differentiation in low-dimensional spaces.

### 3.3. Effect of the number of neurons in the hidden layer and output layer on SVM classification

To further determine the appropriate size of the hidden layer and output layer of the autoencoder, we tested the effect of the number of neurons in the hidden layer and output layer on the SVM classification results for the above combination. In this test, we also use 10-fold cross-validation, and for each autoencoder structure, we encode all the original data using the autoencoder after training, then train 6 SVM classifiers using the encoded data, which do not use the kernel trick and have a C-value of 1. Finally, the final prediction of the algorithm is determined using the voting results of all SVMs on the encoded data. We use the prediction error rate as a measure of the algorithm’s classification performance, which is expressed as the proportion of samples with incorrect predictions for all samples. The test results are shown in Figure 6B.

The results show that the number of hidden layer neurons shows a negative correlation with the recognition error rate. When the hidden layer is 30 neurons or more, the recognition error rate decreases and then increases with the increase of output layer size, and as the output layer size continues to increase, the error rate tends to saturate. The algorithm performs better with a larger hidden layer and a smaller output layer. As can be seen, the scale of the hidden layer plays a key role in the final performance of the algorithm. At the same time, as we expected, too small or too large an output layer leads to a reduced degree of linear separability after encoding the raw data, which is not entirely determined by the encoding performance of the autoencoder; for example, the autoencoder exhibits a high validation error when the number of neurons in the hidden layer and output layer is 50 and 15, respectively, but performs well in the final classification, and the opposite is true at their number of 60 and 25, respectively, although overall it remains that the lower encoding error leads to better classification performance.

### 3.4. Autoencoder training results

**Figure 7:**
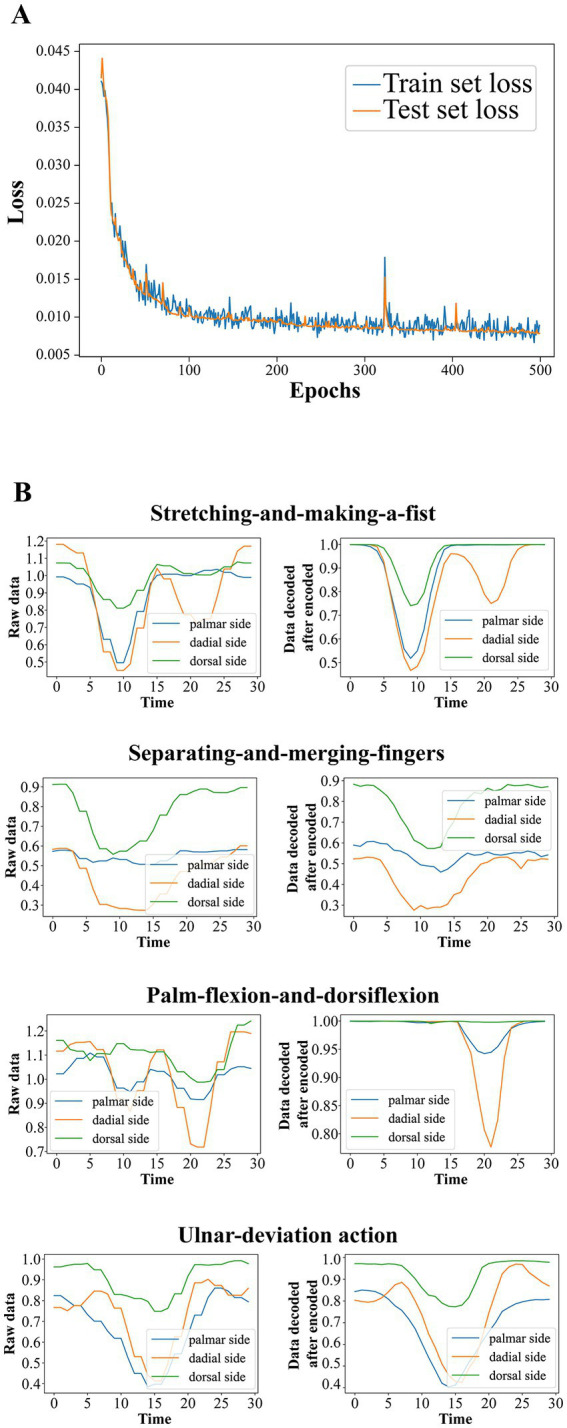
**(A)** The variation of the training set error and the test set error of the autoencoder with the training batch. **(B)** The raw data of the rehabilitation training actions and the corresponding fitted data of the autoencoder first encoded and then decoded.

Considering the computing power of the microcontroller and the generalization capability of the model, we choose a final autoencoder structure with a hidden layer size of 40, an output layer size of 10, the Tanh function as the hidden layer activation function, and no activation function for the output layer. With this parameter setting, we randomly divide the original data into training and test sets according to 8:2, use Adam as the optimizer with each mini-batch size of 200, and select MSE as the loss function. The variation of the training set error and the test set error of the autoencoder with the training batch is shown in Figure 7A. It can be seen that during the training process, the test set and training set errors maintain the same downward trend and have very close values, characterizing the effective extraction of the raw data by the autoencoder. The raw data of the rehabilitation training actions and the corresponding fitted data of the autoencoder first encoded and then decoded are shown in Figure 7B.

### 3.5. Linear separability test after data encoding

**Figure 8:**
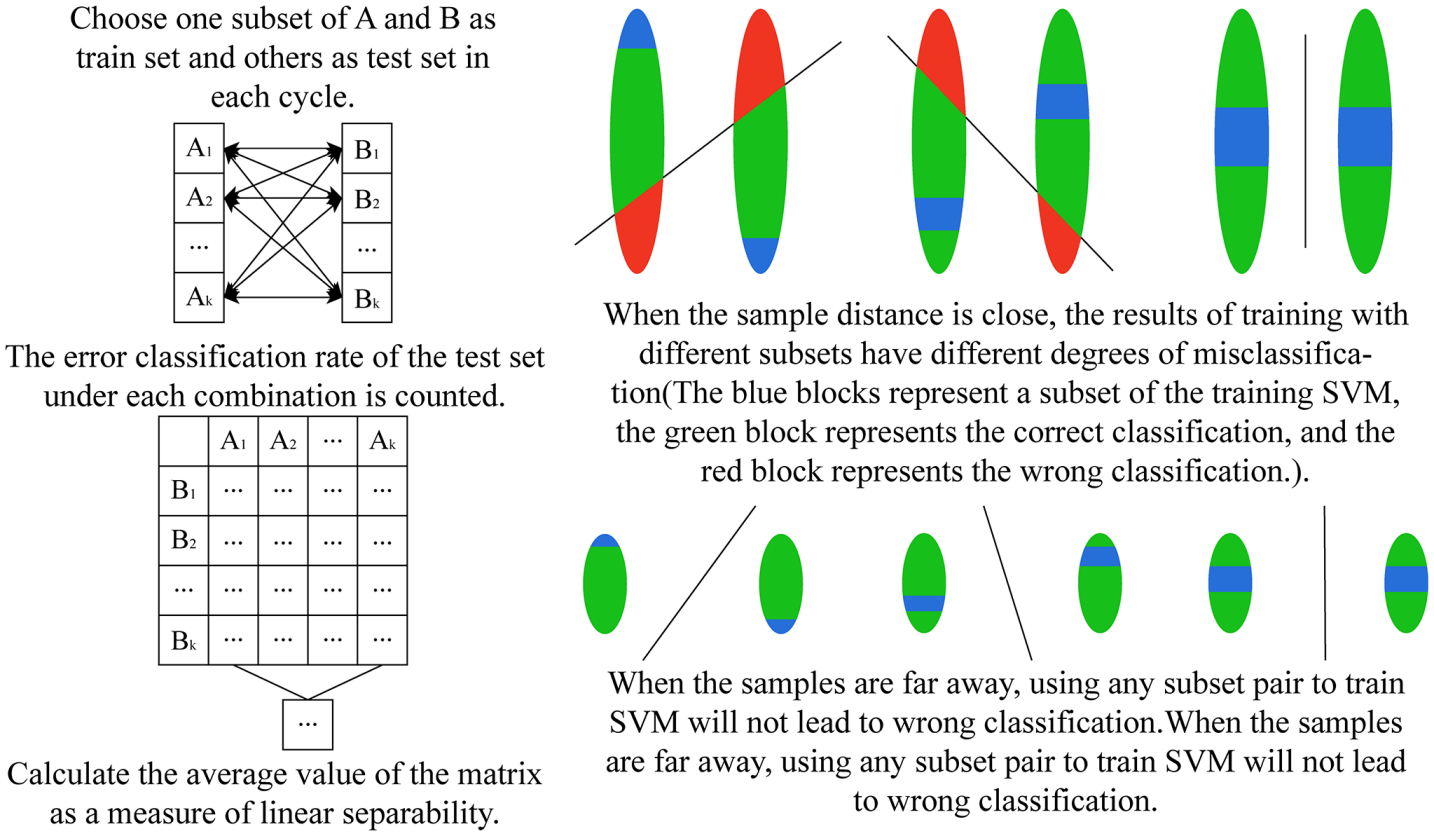
The schematic diagram of generalized k-fold cross-validation.

**Figure 9:**
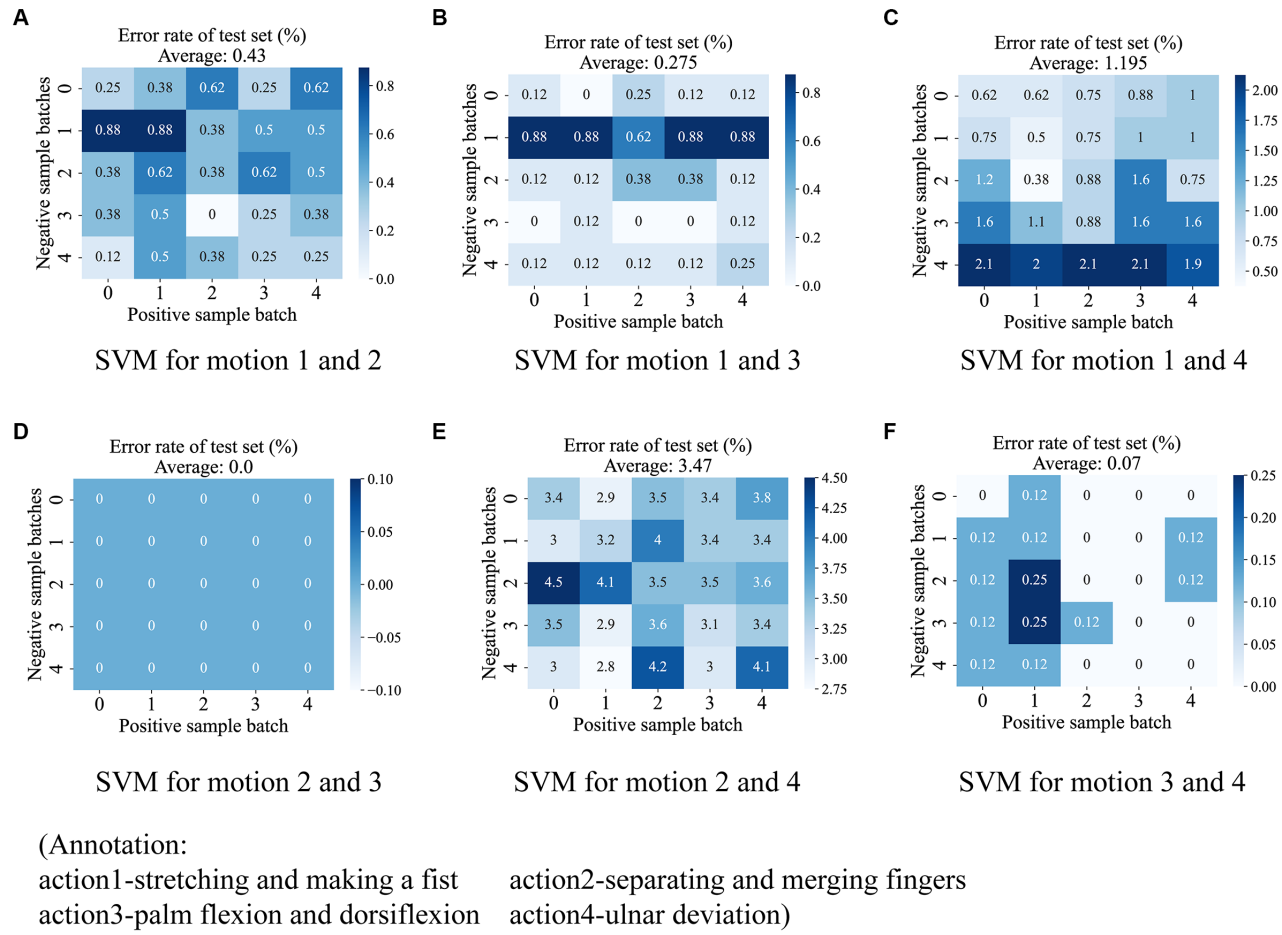
The test results for the six SVM classifiers using the generalized 5-fold cross-validation. The six subplots show the test results for each of the six SVM classifiers, including **(A)** between stretching-and-making-a-fist and seperating-and-merging-fingers, **(B)** between stretching-and-making-a-fist and palm-flexion-and-dorsiflexion, **(C)** between stretching-and-making-a-fist and ulnar-deviation action, **(D)** between seperating-and-merging-fingers and palm-flexion-and-dorsiflexion, **(E)** between seperating-and-merging-fingers and ulnar-deviation action, and **(F)** palm-flexion-and-dorsiflexion and ulnar-deviation action. The results show that there is a maximum linear separability between seperating-and-merging-fingers and palm-flexion-and-dorsiflexion **(D)**, however, there is a minimum between seperating-and-merging-fingers and ulnar-deviation action **(E)**. This is due to the fact that the two actions in the latter have a relatively high similarity in the data change curves.

After the autoencoder training is completed, we encode all the original data. According to our strategy, we are going to use six SVMs to vote on the categories of the encoded samples. Each SVM classifies two of the four classes of actions, such that the six classifiers are denoted as $S_{ij}\left(i=1,2,3,4;j=i,\ldots 4\right)$, denoting the SVMs that classify class i with class j. Therefore, it is necessary to verify the degree of linear differentiability of the coded samples to justify our use of SVM. We propose a generalized k-fold cross-validation to assess the linear separability of the samples. The process is as follows: for the sample sets A and B to be classified, A is labeled as 1 and B is labeled as 0. Each of A and B is randomly disrupted and divided into k subsets as $A=A_{1}+A_{2}+\ldots +A_{k},B=B_{1}+B_{2}+\ldots +B_{k}$. Unlike k-fold cross-validation, we only take a pair of subsets $A_{i},B_{i}$ as the training samples of SVM, and use the remaining samples $A-A_{i},B-B_{i}$ as the test set, and finally calculate the misclassification rate of the test set under each combination and their mean values as the quantified index of the linear separability of the samples. If two sample sets have a high degree of linear differentiability in the sample space, it means that they have a larger distance and indicates that our SVM evaluation made based on the overall sample has a larger confidence level. A two-dimensional schematic of the generalized k-fold cross-validation process is shown in Figure 8. The test results for the six SVM classifiers using the generalized 5-fold cross-validation are shown in Figure 9. The stretching-and-making-a-fist and the ulnar-deviation actions and the separating-and-merging-fingers and the ulnar-deviation actions showed relatively high classification errors, indicating that they were close to each other in the sample space, especially between the separating-and-merging-fingers and the ulnar-deviation actions, which showed relatively high similarity in their pressure curves, leading to large classification errors, while the separating-and-merging-fingers and palm-flexion-and -dorsiflexion actions showed complete classification in all five subsets of the two sample sets, indicating that they were farthest apart in the sample space.

### 3.6. Measured performance of the algorithm after deployment on the microcontroller

We deployed the entire classification algorithm trained on a microcontroller and tested it in practice. In addition to the five persons involved in the training set collection, we called five additional subjects to participate in the actual test, with each subject performing one round of testing. After the subject has tied the wristband and placed the elbow on the table with the arm upright and keeping the palm facing inward, the microcontroller first asks the user to stay relaxed and follow the instructions from the phone for the data acquisition of resting state, followed by the average calculation of the three-channel AD values. Thereafter, the microcontroller determines whether a rehabilitation action is coming according to the above action pre-detection strategy, and if so, classifies the action category, and if it is recognized as a rehabilitation action, sends a response message to the phone. Each subject performed 100 sets of each of the four types of rehabilitation training, with a supervisor counting manually on the side, and finally counting the number of differences between the rehabilitation training actions detected by the microcontroller and the actual actions performed. The confusion matrix was plotted based on the experimental results of each subject, and finally, the accuracy and recall of each rehabilitation training action recognition for each subject were counted.

**Figure 10:**
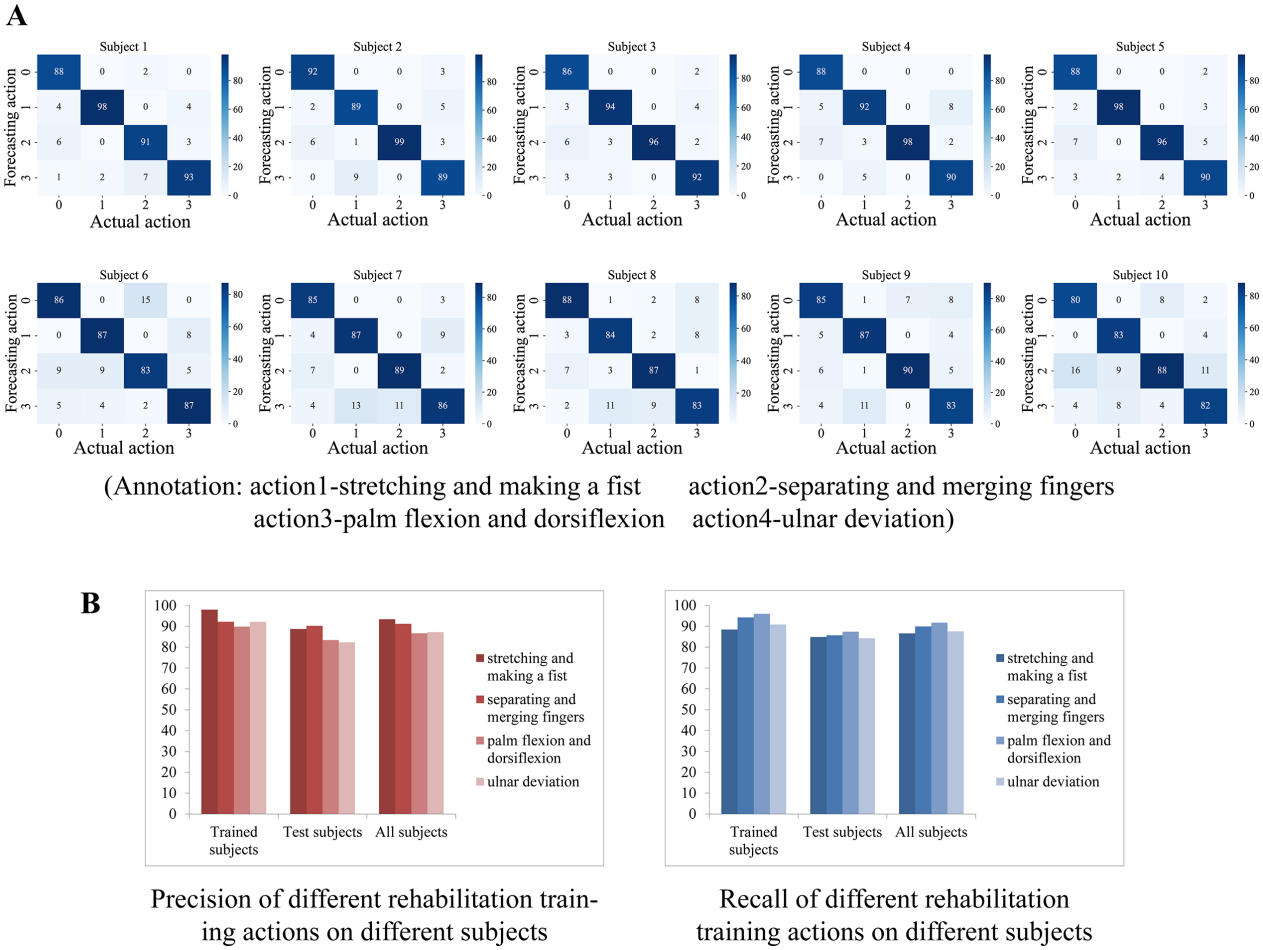
**(A)** The confusion matrix of 10 subjects. **(B)** The result of average precision and recall of the participants who participated in the training set acquisition versus those who did not.

The confusion matrix of 10 subjects is shown in Figure 10A, where the top row shows the test results of subjects who participated in the training set acquisition, and the bottom row shows the test results of subjects who did not participate in the training set acquisition. We counted the precision P, recall R, and f1 scores of each rehabilitation training action for each subject according to the confusion matrix, which was calculated by the following equations


$$P=\frac{TP}{TP+FP}\times 100\%$$

$$R=\frac{TP}{TP+FN}\times 100\%$$

$$f_{1}=2\cdot \frac{P\cdot C}{P+C}$$

For the rehabilitation training action X, TP denotes the number of times the network correctly recognized X, FP denotes the number of times other actions were recognized as X, and FN denotes the number of times X was recognized as other actions or not recognized as any one action. The results show that the test results of the subjects who participated in the training set acquisition were generally good, indicating the effectiveness of the algorithm in classifying the four actions. Subjects who did not participate in the training set acquisition had slightly worse test results than the former but also showed sufficiently high generalization ability, indicating that the algorithm is generalized for feature extraction of the four actions.

The average precision and recall of the participants who participated in the training set acquisition and those who did not participate in the acquisition were counted separately on the four actions, and the results are shown in Figure 10B. On subjects participating in training set acquisition, the recognition accuracy and recall of the four rehabilitation actions were basically above 90% (except for palm-flexion-and-dorsiflexion action with 89.9% accuracy and extension grip with 88.4% recall), with extension grip having the highest accuracy of 98% and palmar dorsiflexion having the highest recall of 96%. On the subjects who were not involved in the training set acquisition, the recognition accuracy and recall rate of the four rehabilitation movements were above 82 and 84%, respectively, with the separating and merging fingers action having the highest accuracy of 90.24% and palm flexion and dorsiflexion action having the highest recall rate of 87.4%. And on all subjects, the average precision of the four rehabilitation movements recognition was 93.38, 91.22, 86.66, and 87.23%, and the average recall was 86.6, 89.9, 91.7, and 87.5%, respectively, and the total precision of the system’s recognition of the four rehabilitation training movements was 89.62%, the total recall was 88.93%, f1 score was 89.27%.

## 4. Discussion

With the development of computers and the Internet, the medical system is gradually showing the trend of intelligence and digitalization. With the support of the Internet and smart devices, rehabilitation treatment is gradually evolving from the previous face-to-face communication between doctors and patients to a new mode of remote monitoring and management. For example, mHealth (Birkmeyer et al., 2021), defined as a medical and public health practice supported by mobile devices, has a large number of applications in clinical diagnosis or advice, improving patient compliance, parameterizing physiological parameters, and providing disease-related education (Rowland et al., 2020). mHealth, as a cell phone APP, can make full use of hardware resources and contains applications such as intelligent intervention, angle measurement (Pourahmadi et al., 2017; Modest et al., 2019; Ochen et al., 2020), intelligent monitoring, and rehabilitation games (Meijer et al., 2019, 2021) in assisting the rehabilitation training of distal radius fractures (Chen et al., 2020). Therefore, it has the advantage of low cost, but its simple architecture dictates that it cannot measure too many physiological parameters, so various terminals are needed to extend its functionality. As a sign of artificial intelligence, virtual reality (VR) technology is also increasingly used in the field of rehabilitation engineering, including assisted rehabilitation training for diseases such as cognitive impairment (Lei et al., 2019), arthritis (Byra and Czernicki, 2020), and chronic obstructive pulmonary disease (Rutkowski et al., 2020), and also in distal radius fractures (Kulkarni and Naqvi, 2021). VR technology in rehabilitation training is basically presented in the form of a serious game, which allows patients to play in the process with less pain caused by the disease and at the same time to carry out effective rehabilitation training, which greatly promotes the enthusiasm of patients in rehabilitation treatment. But playing VR games usually requires an empty space as well as an expensive headset, which makes it seem more appropriate for applications in specialized, centralized retreats rather than for individual users. There are some specialized integrated devices for rehabilitation training, which use surface EMG signals, inertial sensing units, flexible pressure sensors, and other means for physiological information acquisition from the affected area, and based on them for applications in the direction of clinical parameter assessment, movement posture detection, etc. These devices tend to achieve very high accuracy of assessment due to multimodal signal processing and can bring a greater variety of rehabilitation training options to patients. However, their high cost and the inconvenience of wearing them are still the main factors that prevent their popularity.

Our developed wristband for rehabilitation training of distal radius fracture directly detects the force on the palmar side, radial side, and dorsal side of the affected limb by means of a three-channel thin film pressure sensor with tying a rigid magic stick band around the outside. After the sensor is connected to the circuit, the rehabilitation training movement detection of the distal radius fracture recovery period can be performed. The measured data showed that the system achieved an f1 score of 89.27% for the recognition of the four rehabilitation actions. The demands placed on splint tightness in patients with distal radius fractures necessitated the use of a pressure sensor to quantitatively assess splint tightness. Pressure sensors can be categorized into piezoresistive, capacitive, optical fiber, resonant, and piezoelectric types based on different principles (Song et al., 2020), and all of them can be fabricated with very small dimensions in the medical field (Chau and Wise, 1988; Kalvesten et al., 1998; Nemani et al., 2013). Due to the need for miniaturization of the system, we have abandoned the use of bulky sensors such as weighing sensors, despite their high accuracy. There are devices known as intelligent splints that are effective in maintaining pressure on the affected area for immobilization. Most of them use airbags for pressure regulation and quantify the tightness based on air pressure sensors, and this type of research is mainly taking place in China. However, none of the sensor arrangements in these systems directly detect the pressure at the fracture point, so we rule out this option as well. Taking all factors into account, we decided that flexible thin-film pressure sensors were the sensors that best met our requirements, as they are small enough that can be easily placed between the skin and the splint without the negative effects of splint immobilization. Flexible pressure sensors have gained wide application in wearable smart devices due to their high flexibility, high sensitivity, and small size (Xu et al., 2018; Huang et al., 2019). We chose a thin-film pressure sensor with a well-established market, costing less than 3 RMB. After the pressure calibration test, the results show that in our pressure acquisition circuit, the accuracy of the sensor is controlled at ±20%, which meets the accuracy standard of the sensor itself, indicating that our acquisition circuit is effective. It should be noted that the accuracy of our thin-film pressure sensor is far less than that of an accurate weighing sensor, but it is accurate enough to meet our splint pressure monitoring needs, and at the same time it is extremely low-cost, which makes it ideal for the Chinese market. The algorithm uses the average AD values of the initial relaxation state as the data benchmark, and the network input data of the training actions is the ratio of the original AD values relative to the data benchmark. Since each user wears the wristband with different degrees of looseness, this facilitates data normalization and thus improves system robustness and generalization. We designed an action pre-detection strategy. Some rehabilitation actions are divided into multiple phases, for example, stretching-and-making-a-fist action includes two phases, stretching and fisting. Since the data window uses sliding detection, in order to prevent data from the first stage from being passed into the classification algorithm once it enters the window, the strategy determines whether the beginning of the action data has moved close to the head of the window queue by judging the volatility of the data in the first third of the window. This strategy ensures that each window of data that enters the network is a complete rehabilitation training action. At the same time, switching between different rehabilitation actions can lead to strong changes in the pressure data, for example, the initial hand gesture with a relaxed hand in the stretching-and-making-a-fist will result in a relatively low-pressure value, while the initial hand gesture with a tight hand in the separating-and-merging-fingers action will result in a relatively high-pressure value. Switching between these two actions produces a rising or falling slope of the pressure value signal. To prevent passing this switching process into the classification algorithm, the action pre-detection strategy excludes this state switching process by calculating the difference between the start and end values of the window pressure data, so that a complete rehabilitation action signal can be initially filtered by this strategy. In the classification algorithm, we first obtain the feature information after dimensionality reduction from the original action data by an autoencoder and then use six SVM linear classifiers to vote on the samples two by two, and the final prediction with the highest number of votes is used as the final classification output. We tested the linear separability of different action-coded data using a generalized k-fold cross-validation method, and the results show that the action-coded data of any two have good linear separability, proving the effectiveness of using the SVM linear classifier. In the actual test, the results showed that the system achieved a classification f1 score of 85.84% for subjects who did not participate in the training set acquisition, which shows the effectiveness of the system operation.

In addition, the rehabilitation wristband can be used independently in a variety of splinting (small splints, casts, thermoplastic splints, etc.) immobilization situations in the early stages of fracture patients. Its pressure sensor can accurately assess the degree of splint tightness by direct contact with the affected limb, which can largely reduce the dependence of splint fixation on physician experience. This gives the wristband the flexibility of application as it can alert users to the loosening of the splint during fixation (Naqvi, 2022). Through the Bluetooth interface of the rehabilitation training wristband, a variety of rehabilitation training software can be developed based on it, such as a database for recording and managing patients’ daily rehabilitation training, and serious games for improving patients’ motivation in rehabilitation training.

## Data availability statement

The original contributions presented in the study are included in the article/Supplementary material, further inquiries can be directed to the corresponding authors.

## Author contributions

XS and GZ designed and planned the study concept. QZ and ZX completed the system construction and algorithm and design drafted the manuscript. XC provided medical guidance. All authors jointly revised the content of the study and agreed to submit the manuscript.

## Funding

This work was supported by Science and Technology Program of Suzhou, China (No. SYG202019) and Science and Technology Program of Jiangsu Province, China (No. BE2021662).

## Conflict of interest

The authors declare that the research was conducted in the absence of any commercial or financial relationships that could be construed as a potential conflict of interest.

## Publisher’s note

All claims expressed in this article are solely those of the authors and do not necessarily represent those of their affiliated organizations, or those of the publisher, the editors and the reviewers. Any product that may be evaluated in this article, or claim that may be made by its manufacturer, is not guaranteed or endorsed by the publisher.
