# Supplementary figures and images for: Wearable rehabilitation wristband for distal radius fractures
Source: Front Neurosci. 2023 Sep 14;17:1238176. doi: 10.3389/fnins.2023.1238176 (PMC10536142; doi:10.3389/fnins.2023.1238176)

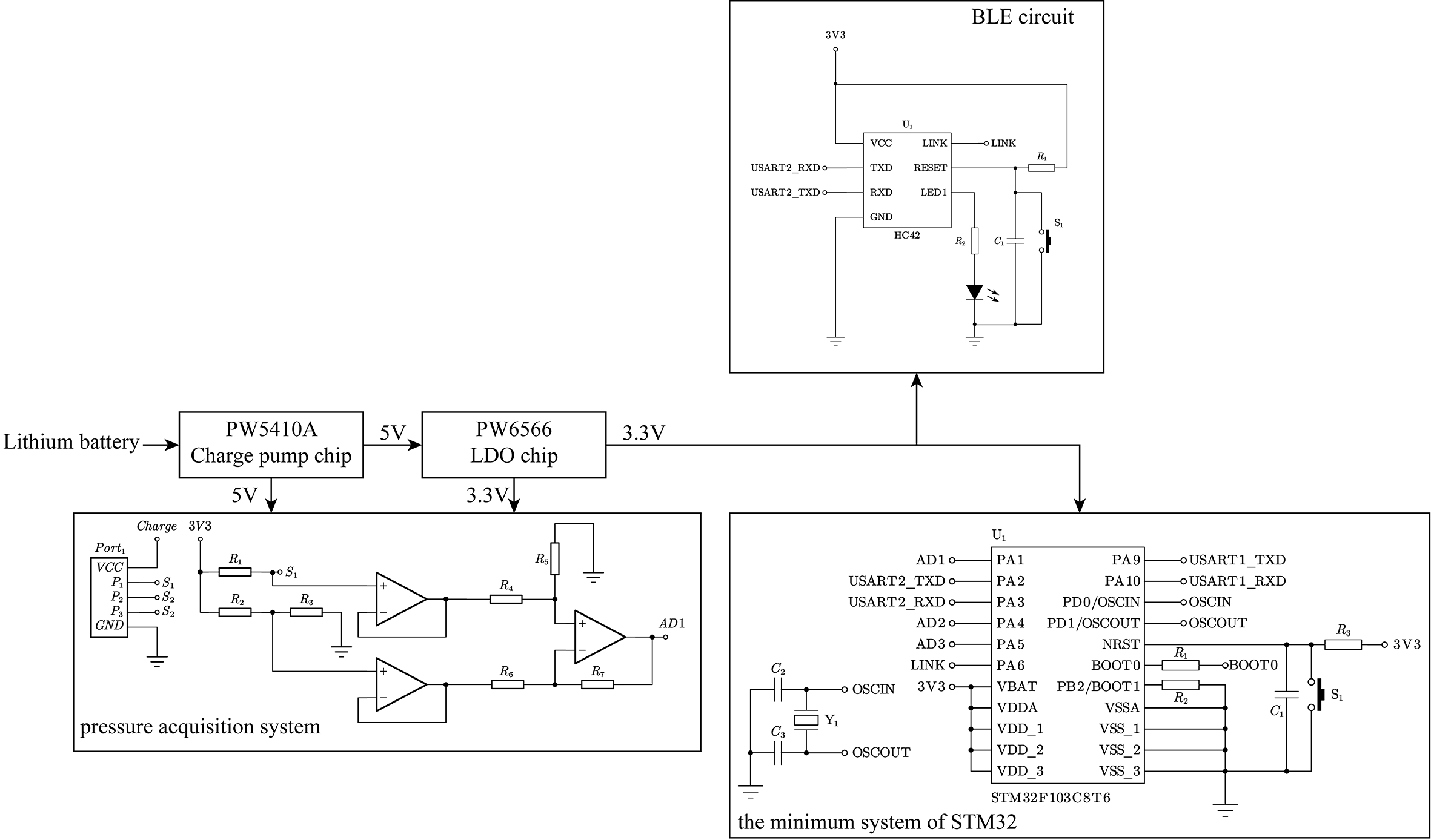

Supplement: Supplementary file 2 [file Image_1.TIF]

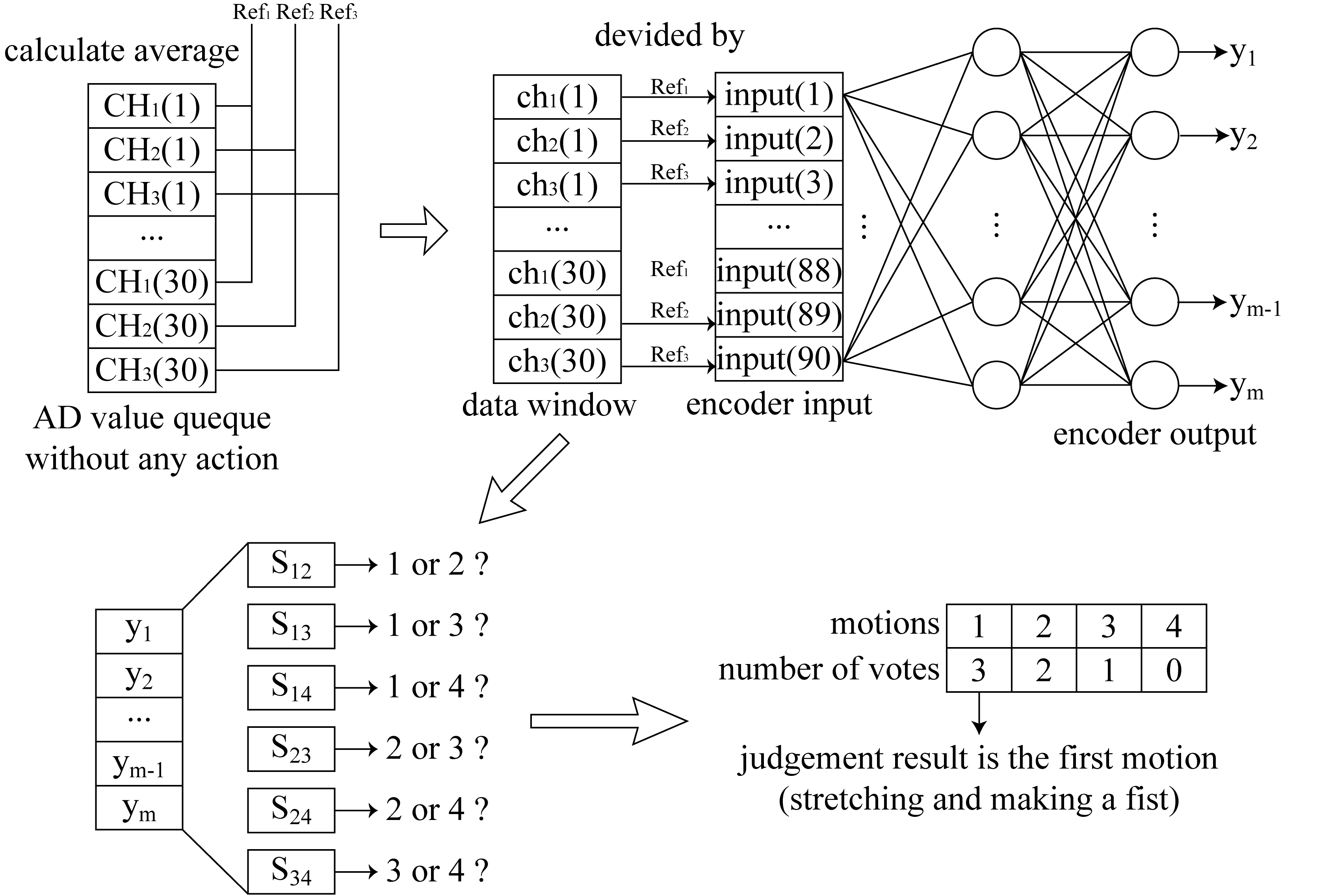

Supplement: Supplementary file 3 [file Image_2.TIF]
